# Salt an Essential Nutrient: Advances in Understanding Salt Taste Detection Using *Drosophila* as a Model System

**DOI:** 10.1177/1179069518806894

**Published:** 2018-11-21

**Authors:** Shivam Kaushik, Rahul Kumar, Pinky Kain

**Affiliations:** 1 Department of Neurobiology and Genetics, Regional Centre for Biotechnology, NCR Biotech Science Cluster, Faridabad, India; 2 Department of Biotechnology, Maharshi Dayanand University, Rohtak, India

**Keywords:** Taste, *Drosophila melanogaster*, neural circuits, salt, gustatory neurons, brain

## Abstract

Taste modalities are conserved in insects and mammals. Sweet gustatory signals evoke attractive behaviors while bitter gustatory information drive aversive behaviors. Salt (NaCl) is an essential nutrient required for various physiological processes, including electrolyte homeostasis, neuronal activity, nutrient absorption, and muscle contraction. Not only mammals, even in *Drosophila* melanogaster, the detection of NaCl induces two different behaviors: Low concentrations of NaCl act as an attractant, whereas high concentrations act as repellant. The fruit fly is an excellent model system for studying the underlying mechanisms of salt taste due to its relatively simple neuroanatomical organization of the brain and peripheral taste system, the availability of powerful genetic tools and transgenic strains. In this review, we have revisited the literature and the information provided by various laboratories using invertebrate model system *Drosophila* that has helped us to understand NaCl salt taste so far. We hope that this compiled information from *Drosophila* will be of general significance and interest for forthcoming studies of the structure, function, and behavioral role of NaCl-sensitive (low and high concentrations) gustatory circuitry for understanding NaCl salt taste in all animals.

## Introduction

Dietary sodium is consumed as a common salt—sodium chloride (NaCl). Sodium, which is present in dietary table salt, is an essential nutrient required for many physiological processes including electrolyte homeostasis, nutrition absorption, maintenance of cell plasma volume, acid-base balance, transmission of nerve impulses, and normal cell physiology. Sodium contributes to the establishment of the membrane potential of most cells and plays a direct role in the action potential required for the transmission of nerve impulses and muscle contraction.

Sodium is a mineral that occurs naturally in foods like flour, mushrooms, celery, beets, and milk and is added in ionized form to table salt (40% sodium and 60% chloride). Packaged and prepared foods like canned soups and frozen eatery items often have added salt during their processing as a measure of preservation. The presence of salt makes food more palatable than the same food with no salt. According to World Health Organization (WHO) details, people in various parts of the world are consuming too much salt in their diets—on an average 9 to 12 g/d which is much more than WHO salt intake guidelines (less than 1500 mg/d, ie, 0.75 teaspoon—3.75 g/d should be consumed). Shown by many groups, extreme intake of salt results in various health issues and causes hypertension, strokes, stomach cancer, osteoporosis, autoimmune diseases, kidney stone, water retention, and bone weakening. Lack of dietary salt intake is also associated with health problems like low blood pressure. Hence, an appropriate amount of salt should be consumed for proper functioning of our body parts and to live longer and healthier.

It is ironic that despite the high incidence of cardiovascular diseases, stroke, elevated blood pressure, and high hypertension-related mortality, we currently do not fully understand the molecular and cellular mechanisms by which low and/or high salt concentrations are perceived or differentially encoded throughout peripheral tissues and in the brain. Furthermore, it is not clear how low or high salt dietary salt intake influences complex feeding behaviors. More research is required in this direction.

This review highlights the studies within *Drosophila melanogaster* that have begun to shed light on the mechanisms of salt detection, how salt influences feeding behaviors, and the influence of salt on other physiological functions. The findings from these studies hold potential to help us understand similar mechanisms that exist in higher order species and may therefore lead to the identification of targetable pathways in human disease.

## *Drosophila* Taste System

Food palatability, or how food tastes, is the main driving factor for initiating a bout of feeding. Like mammals, insects can detect and discriminate among different gustatory stimuli, such as sugars, bitter substances, and various salt concentrations, which induce an attractive or a repulsive response in behavioral tests. Gustatory signals have been shown to play vital roles in controlling behavior, such as searching for food or finding sexual partners.^1^ *Drosophila* is among the most highly studied genetic model systems for investigating feeding behaviors and peripheral and central taste coding. A total of 60 genes in the gustatory receptor (GR) gene family encode 68 receptor proteins.^2-4^ A number of studies within the past decade have focused on understanding the molecular and cellular mechanism by which different taste modalities (i.e. sweet, bitter, water, salt) are perceived in *Drosophila*. The response profiles of gustatory neurons have been described, revealing their specificity, tuning breadth, and behavioral roles,^5-7^ highlighting the complexity of stimulus representation in the taste system as a whole. These findings have invited experiments to probe the extent to which the fly can use this information for both hard-wired and experience-guided behaviors.^8^ Unexpected interactions between aversive tastants and appetitive neurons have emerged by performing functional analysis of taste neurons and have revealed at least 2 distinct mechanisms by which sweet taste neuron activity is inhibited by bitter tastants.^9,10^

**Figure 1.**
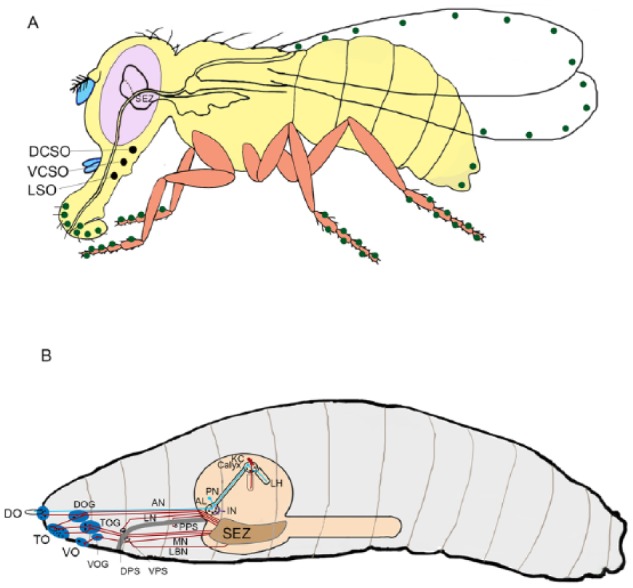
Gustatory system of *Drosophila*. (A) Cartoon showing the presence of GRs on various taste organs (labellum, legs, anterior margin of wings, pharynx, and genitalia) in adult fly as green spots. The pharyngeal organs are LSO (labral sense organ), VCSO (ventral cibarial sensory organ), and DCSO (dorsal cibarial sensory organ). The peripheral taste neurons from various organs terminate in the brain in specialized areas called SEZ. (B) Chemosensory system of *Drosophila* larva. The 3 external chemosensory organs, the dorsal organ (DO), terminal organ (TO), ventral organ (VO), and the dorsal, ventral, and posterior pharyngeal sense organs (DPS, VPS, PPS) include mainly gustatory sensilla. The sensory neurons cell bodies are collected in ganglia below each sense organ (DOG, TOG, VOG). Some neurons innervating the TO are also located in the DOG. Olfactory receptor neurons project into individual glomeruli of the larval antennal lobe (AL), which are interconnected by local interneurons (IN). Projection neurons (PN) link the AL with 2 higher olfactory centers, the mushroom body (MB) calyx and the lateral horn (LH). An intrinsic MB Kenyon cell (KC) is shown in red. GRN afferents (brown) extend via 4 different nerves to the SEZ. The pharynx is shown in gray. Source: Adapted from Gerber and Stocker.^13^ AN indicates antennal nerve; LN, labral nerve; MN, maxillary nerve; PPS, posterior pharyngeal sensillae; LBN, labial nerve.

Expression profiles of GRs in various taste organs such as the labellum, legs, anterior margin of wings, pharynx, genitalia, and in internal organs including intestine in adult *Drosophila*^11,12^ have been shown by various laboratories. (Figure 1A). GRs also express in organs of *Drosophila* larvae such as the terminal organ and pharyngeal sense organs^14^ (Figure 1B) and are shown to be involved in sensing sweet and bitter compounds.^15-17^ *Drosophila* larval olfactory and gustatory chemosensory organs located on the head surface are dorsal organ (DO), terminal organ (TO), and ventral organ (VO), and 3 pharyngeal organs^12^ (Figure 1B). Olfactory receptor neurons (ORNs) located in the DO project into glomeruli of the antennal lobe (AL), whereas gustatory receptor neurons (GRNs) project via 4 different nerves to the subesophageal zone^12^ (SEZ) (Figure 1B). During metamorphosis in *Drosophila*, almost the entire larval peripheral nervous system disappears to be reconstructed into the adult peripheral nervous system.^18^ The pharyngeal sensory neurons in *Drosophila* have been demonstrated as the primary gate keepers of the taste system^1,19,20^ (Figure 1A) are an exception to this rebuilding during metamorphosis.^21^ It is not clear though what this means for the development and physiology of the organism. More detailed functional analyses are required to understand the involvement of the pharyngeal sensory neurons of the labral sense organ (LSO), the ventral cibarial sensory organ (VCSO), and of the dorsal cibarial sensory organ (DCSO). Identifying chemicals these neurons detect may provide insight to understand why these neurons are maintained throughout development and if the function is conserved during larval and adult stages.

In adult *Drosophila*, 3 sensory organs exist in the pharynx: LSO, VCSO, and DCSO.^11,22^ The DCSO and VCSO occupy the dorsal and ventral parts of the cibarium, and the LSO is located near the labellum (Figure 1A). The pharyngeal sense organs are situated in an anatomically ideal position to act as additional regulators of feeding that is between the external sensory system to sense the quality of food, and the post-ingestive internal nutrient sensing system which determines whether to continue or stop feeding. Adult GRNs that express sugar receptors promote and maintain feeding,^7^ while bitter sensing by larval pharyngeal GRNs inhibit ingestion.^15^ Although emerging evidences suggest that the pharyngeal sense organs indeed regulate feeding, but much is still undetermined.

## Salt: An Unavoidable Taste

Among the various taste modalities, NaCl plays an important supportive roles in a multitude of physiological processes, including neuronal function. Both sodium and chloride must be ingested and maintained at proper concentrations throughout the body by homeostatic mechanisms that ensure ideal osmolarity. Therefore, consumption of just the right amount of salt is required; one may expect the behavioral effects of salt being tightly regulated according to concentration. Indeed, the appetitive responses to low salt gradually turn into aversion as the concentration increases,^23-25^ and these opposing behavioral responses are generated by discrete molecular and cellular processes.

As mentioned elsewhere not only sodium, potassium, calcium, and magnesium are other important nutrients required in our body and act as electrolytes. Chlorine plays a fundamental role in digestion by helping to maintain acid-base balance, and it also aids in the absorption of potassium. Magnesium chloride is consum.^29^ed as a supplement version of magnesium, a mineral found naturally in the body, and is essential for muscle and nerve function as well as heart and bone health. Magnesium chloride supplements are not necessary unless one is deficient for the mineral. Salts like potassium chloride provides similar properties like NaCl but with several unwanted side effects, of which the most important have relatively offensive side tastes: bitter, acrid, and metallic. The other salt is calcium chloride which provides a small amount of dietary calcium important for maintaining strong bones and plays a role in nerve impulse and muscle function. Sodium bicarbonate (baking soda) is similar to that of table salt for maintaining optimal health and helps in neutralizing stomach acids. When these salts are consumed sodium, chloride, and bicarbonate become electrolytes that carry electrical impulses in our body.

## Role of DEG/ENaC Channels in Salt Taste

Like other taste modalities, the gustatory system of animals recognize NaCl and other salts. This chemical sensory modality allows animals to detect and ingest salt, discriminate between different salts and allows them to avoid high salt concentrations that can have deleterious effects on the body. It has been shown in different organisms that moderate and low concentrations of Na^+^ salt (<100 mM) are appetitive and are mainly sensed by amiloride-sensitive epithelial sodium channel (ENaC).^26^ ENaC-KO mice lack responses in low salt (amiloride-sensitive) pathway.^26^

Amiloride-sensitive degenerin/epithelial Na+ channels (DEG/ENaC) form constitutively open and cation selective pores.^27^ The involvement of the DEG/ENaC channels in sensing salt taste as receptors are supported by the localization of the α, β, and γ ENaC subunits in mammalian taste receptor cells, as well as by the observations that aldosterone increases the apical localization of these subunits.^28-32^ This is further supported by the observations that amiloride impairs gustatory responses to NaCl.^33,34^ Extracellular amiloride blocks most DEG/ENaC channels.^35^ Although amiloride is not a specific inhibitor of DEG/ENaC channels, it can also inhibit other membrane transport processes. Amiloride-insensitive mechanisms have also been proposed to participate in mammalian NaCl taste and include Na/H antiporters^36^ and cation diffusion through tight junctions.^29^

*Drosophila* genome has a large number of predicted DEG/ENaC genes,^37^ and these channels have been later tested for their role in salt taste. The degenerin epithelial Na^+^ channel gene family, known as the *pickpocket* genes in *Drosophila*, encode subunits of non-voltage gated, amiloride-sensitive cation channels. It has been suggested that DEG/ENaC channels may be formed by homo- or heteromeric arrangements of subunits. Each subunit has 2 transmembrane domains and a large cysteine-rich extracellular loop domain. DEG/ENaC channels are functionally diverse, with roles in fluid and salt absorbance, mechanosensation, and chemosensation. The degenerin/epithelium sodium channels Pickpocket 11(ppk11) and Pickpocket 19 (ppk19) have roles in the detection of sodium and potassium and are also expressed in the tracheal system for liquid clearance.^25,38^ In larva, ppk11 and ppk19 are found on taste sensing terminal organ and in adults they are present on labellum, legs, and wing margins. Both the larval terminal organ and the adult labellum taste bristles consist of bipolar taste receptor neurons surrounded by supporting cells; this arrangement forms a specialized structure containing a pore at its tip.^39^ Thus, the localization of PPK subunits in these specialized sensory neurons position them where they could detect changes in salt concentration. Flies with disruption of PPK11 or PPK19 fail to recognize low concentration of sodium or potassium in water.^25^ Expression of these genes is necessary for the appetitive behavioral responses to low salt, but are dispensable for the aversive responses to high salt.^25^ The evidence provided by Liu et al^25^suggests that the PPK genes are involved in salt taste raises the possibility that salt detection mechanisms are conserved from flies to mammals. A deeper understanding of how salt is detected requires the identification of additional DEG/ENaC subunits and associated proteins (beyond PPK11/PPK19).

## The Low Sodium Permeable Channel Ir76b

**Figure 2.**
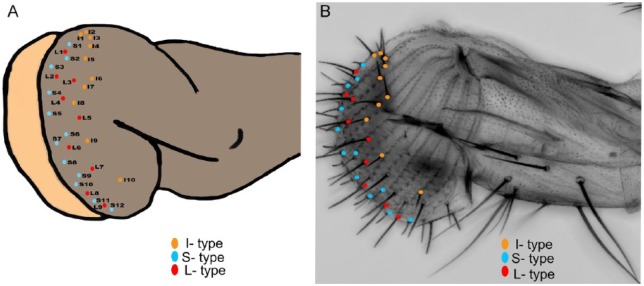
Schematic diagram of the adult *Drosophila* labellum showing sensillar classes (lateral view). (A) Cartoon showing location of the 31 chemosensilla identified by various groups. (B) Confocal image of the labellum showing various taste sensilla types. Anterior is top and dorsal to the right. Color code for taste sensilla: orange (intermediate-I type sensilla), sky blue (small-s sensilla type), and red (large-L type sensilla).

Ionotropic receptors (IRs—comprise a subgroup of the ionotropic glutamate receptor (iGluR) family) have been recognized as critical sensory receptors in insects for detecting environmental stimuli such as chemical compounds,^40,41^ temperature changes,^42^ and humidity.^43^ Recently, it has been established that the salt attractive pathway for low salt relies on a Na^+^ permeable channel ionotropic receptor IR76b, formerly not known to function in taste.^44^ This channel bears no relationship to ENaC channels which are necessary for sensing low salt and showing appetitive responses to Na^+^ at low concentrations in mice.^26^ In particular, IR76b is expressed in L-bristle GRNs of labellum (Figure 2A and B) and is proposed to serve as a functional *Drosophila* counterpart of mammalian ENaC. It has been proposed that some ENaC channels may be constitutively active^27^ leading to depolarization of taste receptor cells following a rise in cation levels at the cell surface. Thus, despite the divergence between fly IRs and mammalian ENaC channels, they may mediate salt taste through similar mechanisms. Zhang et al^44^ have described competition between taste neurons in the S- and L-type taste sensilla (Figure 2A and B) accounts for the bidirectional behavioral responses to salt. At low salt concentrations, the low-salt GRNs dominate over the high-salt GRNs, thereby causing the animals to prefer low salt. Inversely, At high salt levels, the high-salt GRNs overwhelm the low-salt GRNs, resulting in salt rejection. This competition model presented for low and high salt taste detection may represent a widely used mechanism for salt taste coding in other animals, including mammals.^44^ In the same study, GRNs in a few s-bristles have been suggested to act as receptors of high NaCl taste in an IR76b-independent manner, while others have shown cells in L- and i-bristles respond to high NaCl concentrations in an earlier study^45^ (Figure 2). Hence, it has been proposed^44^ that NaCl perception in *Drosophila* adults is determined by a bimodal switch system operating in taste neurons that allows detection of low- and high-NaCl concentrations separately. It has been demonstrated though that the ionotropic channel IR76b is selectively involved in the attractive pathway.

**Figure 3.**
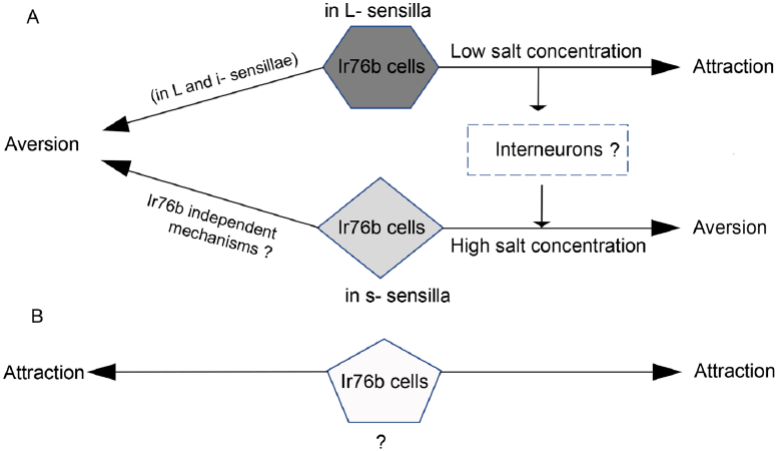
Two different models of feeding preference decision in response to Na+ concentrations (adapted from Lee et al^46^. (A) According to Zhang et al,^44^ *Ir76b* GRNs in large (L)-bristles signal brain for feeding attraction in response to low salt concentrations. At high salt concentrations, the interneurons of unknown identity, may lead to activation of L-bristle GRNs which may in turn aid in robust excitation of the aversive s-bristles GRNs. In this manner, *Ir76b* GRNs in L-bristles, are required for both attraction and aversion to increasing concentrations of Na+. The other possibility would be that the L-bristle GRNs code for repulsion, as opposed to the conclusion by Zhang et al.^44^ The aversion could be also be through GRNs in L and i- sensillae^45^ or through Ir76b independent mechanisms. In any case, there is a possibility that unidentified *Ir76b* cells exist for feeding attraction, as in the absence of the L-bristle GRNs the balance of the whole *Ir76b* cell population move towards attraction (B).

Little is known about the molecular mechanism of high-salt taste in *Drosophila* as in most other animals. More recently, another group^46^ has discovered that suppression of feeding behavior by high sodium-rich food also require *Ir76b* which is necessary for neuronal Na^+^ responses in the s-bristle taste cells besides the previously reported L-bristle GRNs in *Drosophila* labellar gustatory receptor neurons. Together, the results from Zhang et al^44^ and Lee et al^46^ suggest that IR76b plays a central role in gustation of both attractive and aversive Na^+^ concentrations possibly in combination with IRs instructing respective functions^46^ (Figure 3).

Heterologous expression of IR76b in HEK293 cells has shown intrinsic Na^+^ sensitivity of the IR76b protein.^44^ In addition, 2 recent studies demonstrated that IR76b is critical for taste to amino - acids.^47,48^ Such dual responsiveness to salt and amino acids is reminiscent of extracellular Ca^2+^-sensing receptor (CaSR)^49^ and mammalian kainate receptor iGluRs, GRIK1 and GRIK2.^50^ CaSR, a G protein-coupled receptor (GPCR), is activated by another divalent cation Ca^2+^ as well as by L-amino acids. The iGluRs homologous to IR76b seem to be functionally conserved in salt sensitivity, as they bind Na^+^ ions in order to be activated by glutamate. In addition, iGluRs have been reported to be present in taste receptor cells.^51,52^ Thus, the findings that IR76b critically contribute to high-Na^+^ taste suggests the interesting possibility that iGluRs may in part mediate the molecular mechanism of high Na^+^ taste in mammals which has yet to be identified. Thus, these results highlight the complexity of IR76b-dependent Na^+^ gustation in *Drosophila*. Various Gal4 transgenes covering different subpopulations of the IR76b cells would facilitate future studies to further dissecting the cellular substrates for IR76b-dependent Na^+^ feeding behavior.^46^

## The Role of Pharyngeal GR2a

In the vertebrates taste system, taste cells are present not only on the surface of the tongue but also in other organs including the pharynx.^53-55^ The Zebrafish T1R2/T1R3 sweet taste receptors are expressed in the pharynx, the lip and in gill raker.^54^ Although expression of T1R2/T1R3 in the pharynx has not been examined in mammals, it is known that the cranial nerve fibers innervating pharyngeal taste cells respond to sugar.^56,57^ These neurons project to the nucleus of solitary tract (NST) in the hindbrain.^56-58^ The NST also receives peripheral satiety signals transmitted via vagal nerve fibers, indicating that this brain region is involved in many regulatory processes that regulate food intake including the decision to ingest.^58,59^ It has been suggested that due to similarities of insect and vertebrate taste processing systems, interneuron populations in the NST with the physiological properties of cholinergic local interneurons (IN1) neurons identified recently^20^ may exist. Understanding how the pharynx perceives taste would permit rapid evaluation of food intake volume and quality and would provide real-time feedback to the central brain to regulate ingestion in both vertebrates and insects.

Using capillary feeder (CAFÉ) assay and GR expression analysis, recently it has been^1^ reported that a pair of pharyngeal gustatory receptor neurons is involved in feeding inhibition in response to high concentrations of sodium ions and function as modulators of feeding. Molecular genetic tools, RNAi experiments, and mutant analyses have shown that the neurons of gustatory receptor *GR2a* are involved in feeding inhibition. This feeding suppression suggests that *Drosophila* find highly concentrated salty food unappetizing, but is not manifested when flies are starved and hungry suggesting starvation influences feeding preferences. These results provide evidence that factors involved in feeding, function in a context-dependent manner since feeding is influenced by many factors including nutritional status or hunger.^1^ Although data suggest that *GR2a* is involved in the aversion to high salt, it is unclear whether GR2a is a salt receptor per se or a modulator of the response.

## Serrano in the Larval Taste System

Serrano (Sano) is an apically enriched cytosolic protein required for neuronal activity in response to high-salt concentrations. *Sano* coexpress with *GR66a* in 4 gustatory neurons in the terminal organ of third instar larvae.^60^ Absence of the DEG/ENaC channel PPK19 that function in the same set of neurons eliminates the cellular response to high-salt concentrations. Thus, both PPK19 and Sano are required on the larval gustatory neurons for the detection of high-salt concentrations. Disruption of *sano* gene expression in gustatory neurons leads to specific loss of high-salt concentration avoidance behavior in larvae. Inactivation of *sano*-expressing GRNs induces an attraction to high-salt concentrations, suggesting that disruption of only aversive salt pathways determines the opposing behavioral responses to low and high salt.^44^

Previous studies have shown that Sano interacts with other proteins, such as Grb2^61^ (a protein involved in the signaling pathways of tracheal and wing development) and with Epac, (exchange protein directly activated by cAMP) a member of the Rap1 signal transduction pathway. It is involved in cell adhesion and differentiation, as well as in neuronal activity by regulating calcium levels or neurotransmitter release.^62^ The genetic and molecular interactions between Sano and Epac is not well understood and remain to be fully demonstrated by future in vitro and in vivo studies. Such studies will provide new insights into the cellular mechanisms taking place downstream of the DEG/ENaC channels in salt-detecting neurons.

## Post Mating Circuit

An animal’s nutritional requirements change over their life time to meet the nutritional demands and animals display specific behavioral adaptations to increase their intake of particular nutrients they require to maintain the homeostasis. An animal’s specific nutrient intake can be adapted to needs in the current state by need-dependent and need-independent mechanisms.^63^ It has been extensively shown that during reproductive period, females’ nutritional requirements change drastically and they tend to invest enormous resources for their progeny. A specific appetite for sodium increases during the reproductive and lactation phases.^64-67^ For the reproductive success, sodium provides ions required for nutrient balance in newly formed eggs or an increase in total food intake which ultimately contributes to egg production.^68,69^

A study conducted by Walker et al^70^ has provided insights into the physiological regulation of salt intake. In particular they have dissected the feed-forward regulation of sensory processing in *Drosophila* female representing a mechanism through which reproductive state-sensitive circuits modify complex behaviors. Authors in this article have demonstrated that mating induces a salt appetite in *Drosophila* as seen in many other species during reproduction. They have also dissected the neuronal mechanisms through which animal’s reproductive state drives salt appetite which does not require octopamine as needed in post - mating yeast appetite. Data suggests that during the reproductive period, female flies crave more for sodium and their feeding rises more toward it. The increased attractiveness for salt positively affects reproductive output in flies as in many animals. This appetite for salt is induced independently of salt requirements for egg laying and by a feed-forward change in taste processing, driven by a male-derived signal acting on female post - mating circuitry. The authors explain that male derive sex peptides after copulation transfers into the neuronal sex peptide receptor (SPR) and also inhibit the action of downstream sex peptide abdominal ganglion (SAG) neurons, which have a role in increasing egg laying and inhibiting remating. Sex peptides acts on a small set of sensorimotor system. This study highlights the importance of reproduction as a critical modulator of taste processing and brings new insight into the mechanistic basis of this state-dependent nutritional modulation. Future investigations are required to explore to what extent feed-forward regulation is employed to control specific behavioral strategies used to acquire nutrients depending on differential internal state signals.^70^

The flexibility of circuit analysis in *Drosophila* offers unique opportunity to understand the circuit mechanisms through which internal state signals modulate taste processing in the brain, and thus bring about adaptive changes in food preference.^71^ To attain this, mating may modulate the response of sensory neurons to salt taste, as demonstrated in the olfactory pheromone system of moths.^72^ Similarly, GRN responses are shown to be modulated by hunger^73-75^ and the sensitivity of pheromone-sensitive olfactory receptor neurons in mice are modulated across the estrus cycle.^76^ Conversely, the mating state could lead to a combination of modulation at the receptor neuron level and modification of higher order processing. Understanding how alliesthesia is implemented at the circuit level will provide an opportunity to understand how internal state changes affect sensory processing to mediate adaptive behaviors.^70^

## Learning and Memory

The knowledge of salt handling in larva is still very limited. A study conducted by Niewalda et al^77^ has explored salt processing in the larval taste system of *Drosophila*. Their data highlight the behavioral effects of NaCl in choice behavior, feeding behavior, and learning shift from appetitive to aversive as the concentration of salt increases. Regarding feeding behavior, others^45^ have reported that in adult flies feeding is upregulated by salt at 0.1 M but is downregulated by 0.4 M salt, with the strongest “appetizing” effect between 0.05 and 0.1 M. These findings fit reasonably well with Niewalda et al^77^ results in larva and suggest some functional conservation of salt processing between larva and adult. Based on the observation that most pharyngeal gustatory sensory neurons of the larva are retained into adulthood, such conserved function had already been proposed.^21^ Studies conducted by Niewalda et al^77^ has dissociated parametrically the reflex releasing (choice, feeding) from the reinforcing function of salt in terms of their respective dose-effect characteristics: the reinforcing effect shifts by one order of magnitude toward higher concentrations. Interestingly, a similar shift between these 2 kinds of behavioral effects is also found for sugars,^78^ suggesting some degree of generality of such parametric dissociation. Thus, for both salt and sugars, the input pathways for gustatory behavior appear to be more sensitive than the ones supporting gustatory reinforcement.

## Conclusions and Future Prospective

### Understanding higher order low and high salt circuitry in the brain

*Drosophila* are capable of detecting taste modalities that are associated with food acceptance or rejection behavior^79,80^ by various taste cells present on the proboscis, legs, and wings of the adult fly.^11^ The different classes of taste cells include bitter cells marked by the GR, namely, GR66a and sweet cells marked by GR64f.^81-86^ PPK28 ion channel expressing water taste cells^87,88^ activated by low osmolarity and are inhibited by high osmolarity. IR76b-expressing taste neurons respond to low salt.^44^

**Figure 4.**
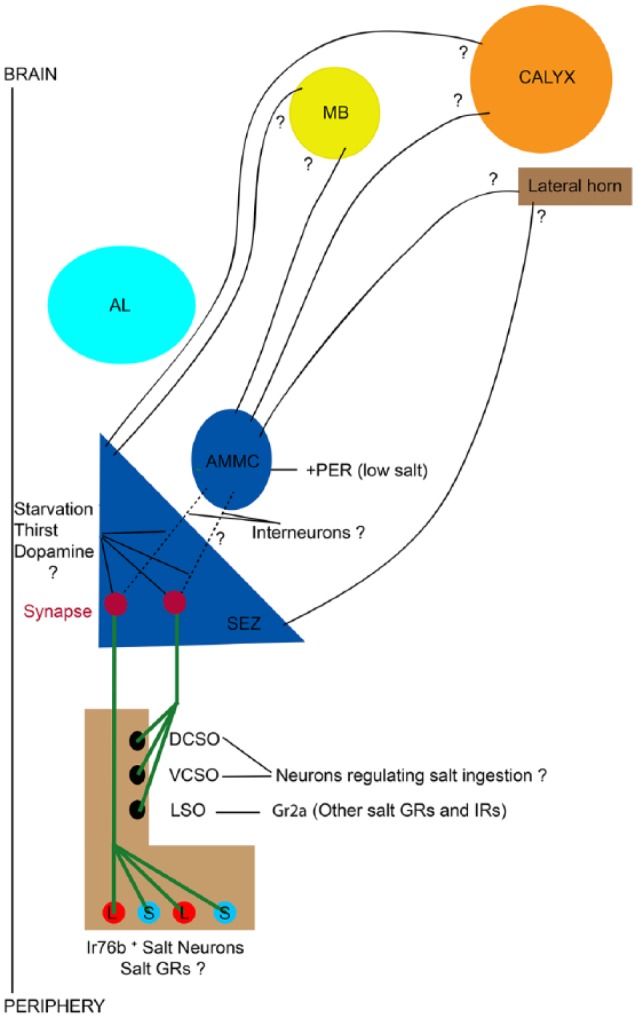
Proposed model. Shown is a schematic illustrating the identified and unidentified components of salt taste circuit in *Drosophila*. IR76b neurons from the periphery send information about salt taste to SEZ. Direct sensors involving gustatory receptors (GRs) for salt remain to be identified. Pharyngeal taste neurons of LSO (Gr2a neurons are involved in feeding inhibition in response to high concentration of sodium ions), VCSO, and DCSO also send processes to SEZ. The identity of other salt receptors and IRs as well as salt taste neurons of VCSO and DCSO are not known yet. Interneurons in the SEZ (black dotted lines) that receive and process gustatory information about salt are largely lacking. Like sweet taste, if any, the role of dopamine signaling in reducing behavioral threshold to salt upon starvation and modulation of feeding responses is not known. It would be interesting to determine if there would be state-dependent (starvation and thirst) alterations in salt taste circuit activity that could lead to more salt eating or eating of high salt concentrations. One needs to verify the possibilities if the information about the starvation state is amplified during the relay to salt second-order neurons or that these neurons may also be targets of signaling pathways that convey information about the starvation state. Role of AMMC as a secondary center for low salt taste as in case of sweet taste is a future question. It is not known where the information from salt taste neurons input upon stimulation of labellum and tarsi taste neurons with low salt concentrations is integrated, either upstream or at second-order neurons. Since salt taste projections to higher brain centers have not yet been characterized, questions regarding the salt circuitry providing gustatory inputs from SEZ or AMMC or both to motor neurons, MB, calyx and lateral horn to control feeding behavior and associations with appetitive and aversive learning remain unaddressed. AL indicates antennal lobe; AMMC, antennal mechanosensory and motor center; DCSO, dorsal cibarial sensory organ; LSO, labral sense organ; MB, mushroom body; PER, proboscis extension response; SEZ, subesophageal zone; VCSO, ventral cibarial sensory organ.

Different peripheral taste neurons expressing taste receptors from the labellum and pharynx target discrete regions of the SEZ,^11,89^ the taste center of the fly brain (Figure 4).^84^ This densely innervated brain structure houses various projection neurons, interneurons, and motor neurons required for taste acceptance and rejection, along with motor circuits that regulate ingestion^73,90-97^suggesting the presence of local circuits in the SEZ that process taste cues from detection to behavior. In addition, neuromodulators, namely, dopamine, serotonin, neuropeptide F, and short-neuropeptide F modulate food intake by altering the activity of sensory neurons that detect food stimuli, or of homeostatic neurons that regulate hunger.^74,75,98,99^ Many tastants that inhibit proboscis extension and feeding act not only via activating bitter taste neurons^5^ but also by inhibiting sweet taste neurons.^9,100^

Only a limited understanding of central neural circuits to examine the modality processing have been attained so far beyond sensory neurons and MNs that induce behaviorally appropriate motor output. Also, the identification of neural circuits that integrate and process information of taste, hunger, and metabolism to control food ingestion remain poorly understood.^58,101^ The presence of a large number of interneurons in the SEZ that appear to respond to gustatory input, it is remarkable that only very few of these have been identified at the cellular level. Few gustatory interneuron types that respond to sweet taste input have been identified in the adult SEZ recently.^1,20,92^ Kain and Dahanukar^92^ reported the first identified second-order sweet gustatory projection neuron (NP1562) that make synaptic connections with GR5a in the SEZ. This set of neurons is unique as they relay sweet taste information from the SEZ to the antennal mechanosensory and motor center (AMMC) in *Drosophila*. These neurons show increased sucrose sensitivity upon starvation and dopamine administration.^92^ Similar to sugars, low salt is innately attractive to most animals. To understand how low salt taste activates appropriate feeding behaviors, higher order neurons in taste circuits must be identified and studied (Figure 4). Proboscis extension can be induced by stimulation of either labellar or tarsal taste hairs in wild-type flies.^101^ Taste neurons originating from these 2 organs target distinct areas in the SEZ.^11,85,86^ Where the information from the 2 organs is integrated is not known either upstream or at second-order neurons (Figure 4).

In a recent report,^102^ large-scale analysis of pan-neural activity in the fly brain suggested that taste modalities in the periphery activate different pathways in the brain. Sweet and bitter tastes are processed by segregated pathways, consistent with labeled line taste processing suggesting strategies that ensure innate responses to essential compounds. Information processing in separate streams is also maintained in the higher brain and is mutually inhibitory. Supporting the studies in the mammalian gustatory system that argues for a modality-specific representation in the gustatory cortex and support labeled line models.^103-105^ These studies suggest that faithful pathways may be a general strategy to process tastes used throughout evolution. The field is still highly controversial though and evidences supporting both “distributed and labeled line model of taste coding” exist and need future examination. Work by Harris et al^102^ has provided a population overview of gustatory processing in the fly that will help to determine the functional role of each neuron during different steps of feeding behavior, the anatomy and connectivity of taste-responsive neurons.

With the exception of 2 identified bitter-sensitive projection interneurons types,^1,106^ information about first-order interneurons that receive and process gustatory information about other tastants categories such as bitter, salt, and water is largely lacking (Figure 4). In a recent study,^97^ the neural connections for bitter taste processing has been investigated. This study has identified a pair of gustatory local interneurons (bGLNs) involved in bitter taste aversion in flies. bGLN dendrites stay in close proximity to axonal termini of bitter-sensory neurons in the SEZ. It is incredible that the bitter taste modality is conserved and evokes aversive behavior in insects and mammals. The identification of bGLN is a significant step towards understanding how bitter taste modalities are processed by the gustatory circuitry in the SEZ of the brain. Whether these or other yet-unidentified SEZ neurons with roles in gustation or feeding are, indeed, post-synaptic targets of the first-order bitter-sensitive interneurons and whether they receive excitatory or inhibitory input from these cells must await further investigation.^97^ Whether the same pathways are involved in detecting high salt, and evoke aversion toward high concentrations is the focus for future studies (Figure 4). Unraveling taste circuits, therefore, will be important not only for understanding how sensory inputs is translated to behavioral outputs but also how taste associations are formed in reward and aversive learning.^8^

### Identifying salt pharyngeal neurons

To control behavioral feeding decisions, animals must simultaneously integrate external sensory stimuli with their internal state.^107,108^ Eat neural metabolic control of eating is regulated both by peripheral sensory detection of food and internal states like hunger and satiety.^109-113^ Dysregulation in these homeostatic systems can lead to metabolic conditions like obesity and other associated health problems. Ingestion is a poorly understood step in feeding behavior. In all animals, the optimization of food intake requires tight regulation of behaviors responsive to food quality and hunger state. After food ingestion, the nutrient sensing signals processed by the intestine take a relatively long period of time to mediate behavioral responses in the brain to change feeding rates.^114,115^

Stimulation of sweet taste neurons in the labellum and legs triggers an extension of the proboscis in starved flies, followed by initiation of food intake.^101,116^ Upon ingestion, the food comes in contact with pharyngeal taste neurons.^11^ Although function of pharyngeal taste neurons is poorly understood, a subset has been shown to regulate sugar ingestion.^7^ Only limited studies have investigated the dynamics of fly feeding using proboscis extension as a proxy for food intake.^73-75,92^ Studies performed on blowflies suggests that food intake is controlled by factors that stimulate ingestion, not the one that act on peripheral taste perception or post-ingestive nutrient-sensing.^116^ Neurons in the fly taste circuit that regulate different aspects of food intake behavior have been identified recently. Neuropeptide F and dopamine signaling enhance the sensitivity of labellar taste sensory neurons in hungry flies and increase their probability of initiating food intake.^73-75^ Perturbation of labellar sweet taste perception does not affect ingestion^7^ suggesting the labellar taste neuron circuitry likely regulates initial food evaluation, but not the later decision to ingest food. Recent work has identified interneurons that regulate the feeding motor program,^90^ GABAergic neurons that suppress nonselective ingestion,^95^ and motor neurons that regulate fluid ingestion.^93^ How these neurons connect taste sensory input to the motor output of ingestion, as well as how they interpret top-down information about hunger state is not known. Yapici et al^20^ propose that 12 cholinergic local interneurons (IN1) participate within this circuit as a key nodes that governs rapid food intake decisions. These neurons in the taste center of the fly brain regulate sucrose ingestion and receive selective input from sweet taste neurons in the pharynx.^7^ The identity of neurons like IN1 that will respond to high concentrations of salt and bitter compounds is still unknown (Figure 4). Analysis of pharyngeal GRN projections also suggests distinct connectivity to higher order neuronal circuits.^19,20^ A recently generated molecular map of pharyngeal taste organs, has opened venues for future investigations to study the roles of pharyngeal taste neurons in food evaluation and in controlling feeding behaviors. Further studies investigating the role of pharyngeal GRNs and pharyngeal taste circuits will provide insight into how internal taste signals are integrated with external taste to control various aspects of feeding behavior (Figure 4).

### Salt representation in higher brain centers

The Mushroom body (MB) is a site for experiential learning in *Drosophila*.^117-119^ The dendrites of the MB principle cells, called Kenyon cells (KCs), receive sparse and random inputs from olfactory projection neurons (PNs). Evidences that the MB processes taste as CS (conditioned stimulus) and US (unconditioned stimuli) comes from behavioral taste conditioning experiments.^120-122^ Pairing sucrose stimulation to the leg (CS) with an aversive stimulus (US) causes short-term inhibition of proboscis extension in the proboscis extension response (PER) assay. Such learned behaviors requires the MB, but the neural processing in the MB that underlies taste conditioning is unknown. The salt taste projections to higher brain centers have not been characterized yet, therefore questions regarding the salt circuitry providing gustatory inputs to the MB remain unaddressed (Figure 4). However, a study^122^ provides direct evidence of multimodal inputs into the MB, with different representations for tastants of different modalities and different representations for different taste organs widening our understanding of the neural coding underlying conditioned learning and providing a basis for examining taste circuitry in the higher brain.

Work performed in rats with salt has provided information about how predictive evaluation can be strongly changed by internal nutrient deficits. Trained rats avoid a metal lever paired with aversive salt concentrations and avidly approach the same lever when sodium is deprived.^26,123^ Establishing similar paradigms in *Drosophila* could be equally informative.

### State-dependent alterations in the salt taste circuit

Peripheral taste processing and the regulation of hunger states in vertebrates have been intensively studied. It has been shown that activation of sweet cells promotes food acceptance in hungry animals, while activation of bitter cells stimulates food avoidance.^124,125^ Neurons in the hypothalamic neuroendocrine circuits express proopiomelanocortin (POMC), agouti-related peptide (AgRP), and melanocortin receptor (MC4R) that coordinate ingestion in response to the hunger state of the animal.^126-129^ The mechanisms controlling taste and food intake in insects are remarkably similar as of vertebrates. Recent evidence in *Drosophila* suggest an increase in dopamine signaling enhancing the sensitivity of sweet gustatory project neurons (NP1562 neurons) to sucrose.^92^ Previously, it has been shown that starvation leads to increases in sucrose-evoked electrophysiological^130,131^ or calcium activity in *GR5a*^+^ taste neurons.^74^ It would be of interest to determine if there are state-dependent alterations in salt taste circuit activity that could lead to more consumption of salt like sugar, or consumption of higher salt concentrations (Figure 4). One needs to verify the possibilities if the information about starvation state is amplified during the relay to salt second-order neurons or if these neurons may also be targets of signaling pathways that convey information about the starvation state. How physiological state like hunger or adaptation to high salt act on these neurons that allows eating of high salt (aversive) concentrations in humans is a subject for future investigations.

### Ir76b and other mechanisms for low salt detection

There are at least four kinds of chemoreceptors that function in the GRNs of D melanogaster including GRs, IRs, TRP (transient receptor potential) channels, and PPKs. The molecular identity of mammalian taste receptors (TRs) and insect GRs are very different. Taste receptors are GPCRs, but GRs are ion channels. The difference of GRs can be used to target insects while having minimal effects on humans. The other sensors such as TRPs, IRs, and PPKs are somehow conserved in vertebrates and invertebrates. Studies on the role of ionotropic cation channels in *Drosophila* taste recognition and regulation of attraction and avoidance behavior in taste are becoming more visible recently. The role of limited IRs in the taste system including IR76b, IR25a, and IR62a have been discovered recently. ^132^ and the function of other IR proteins in the taste system is still unexplored. It will be interesting to determine whether IRs function in concert with GRs, or whether they independently recognize other classes of tastants. The role of TRP channels, PPK proteins, or direct sensors involving GRs for detecting salt remain to be identified as well.^8^

The behavioral valence to salt depends on its concentration. Low salt is appetitive, whereas high salt is aversive. “Salt” neurons in L-type labellar sensilla display peak responses to around 100 mM NaCl and evoke appetitive behavior. IR76b-positive salt neurons show an attractive response to low salt and confer salt sensitivity when expressed in sweet neurons.^44^ Expression of IR76b has been observed in non-salt gustatory neurons, and in several classes of olfactory neurons that are likely salt insensitive.^40^ Whether, and how IR76b channel activity is gated in these neurons remains to be determined.

Similar to adult flies, the high salt responses are genetically separable from low salt response in larvae. Salt taste in larvae appears to be dependent on *ppk* genes. Both *ppk11* and *ppk19* genes are required for behavioral attraction to low salt and salt sensitivity in the terminal organ.^25^ As in adult flies, behavioral aversion to high salt relies on *ppk19* and *serrano*.^60^ The *ppk* genes may not be necessary for salt taste in the adult fly, raising questions about why there exist 2 different molecular mechanisms for low salt.^8^

### Understanding the role of sugar, bitter, and sour gustatory pathways in salt detection

Peripheral gustatory neurons in adult *Drosophila*^84^ express different members of the GR gene family and can be activated by salt with low threshold and by sugars (GR5a) and by salt with a high threshold and by bitter substances (GR66a). Additional studies are required to understand if such mechanisms operate in the same set of taste neurons that sense sugars and bitter compounds. Such studies will also shed light on mechanisms where loss of neuronal activity in sweet and bitter neurons can modulate behavioral valence to salt.

The taste of highly concentrated salt is shown to be aversive in animals ranging from nematodes to rodents.^77,133,134^ Even humans find high salt concentrations to have a bitter taste, therefore the aversive response to high salt concentrations may be more complex than previously thought. Electrophysiological studies performed on *Drosophila* adult taste sensilla have revealed that low- and high-NaCl concentrations are detected by 2 distinct gustatory neurons.^45,135^ L1 neurons respond to salt with a low threshold between 0.01 and 0.05 M, whereas the threshold for L2 neurons is about one order of magnitude higher concentration.^135^ In addition, the *dpr* locus (for defective proboscis extension response), a member of the Ig superfamily, has also been shown to be required for the aversive response to high-salt concentrations in adult flies.^136^

Recently, it has been shown^134^ that high-salt recruits 2 primary aversive taste pathways in mice by activating the sour and bitter taste-sensing cells. Genetic silencing of sour and bitter pathways eliminates behavioral aversion to high concentration of salt, without impairing salt attraction. Mice devoid of salt-aversion pathways exhibit unimpeded, continuous attraction even to exceedingly high concentrations of NaCl. These data suggest that “co-opting” of sour and bitter neural pathways evolved as a means to ensure that high levels of salt reliably trigger robust behavioral rejection, thus preventing its potentially detrimental effects in health and well-being. It would be interesting to dissect if similar pathways are involved in insects. As understanding of how low and high salt concentrations are differentially encoded is still unclear, future studies using specific inhibitors and activators of individual pathway should help address the contributions of the ENaC, T2R, and PKD2L1-expressing taste cells to human salt taste perception. These studies may serve as a catalyst for the development of selective receptor cell modulators to help control (and even satisfy) the strong appetite of the Western world for a high-salt diet, but without the potential ill effects of too much sodium.^134^
